# A Case of Chronic and Latent Pleura-Pneumonia, in Which Nearly All the Functional Signs Were Absent

**Published:** 1841-08-07

**Authors:** Richard A. Sale

**Affiliations:** Bedford County, (Va.,)


### MEDICAL EXAMINER.

DEVOTED TO MEDICINE, SURGERY, AND THE COLLATERAL SCIENCES.

No. 32.] PHILADELPHIA, SATURDAY, AUGUST 7, 1841. [Vol. IV.

### ORIGINAL COMMUNICATION.

*A case of chronic and latent Pleuro-pneumonia, in which nearly all the functional signs were absent.* By Richard A. Sale, M. D.

A coloured man, aged about 40, a blacksmith by trade, of rather intemperate habits, but originally robust, and of a good constitution, complained about three months ago of debility, which, continuing, caused his master to request my attendance.

On visiting him I was very particular and minute in my inquiries as to the locality of his disease. All I could learn from him was, that he felt general debility and had been subject to attacks of colic. He further stated that previously he had a cold attended with a slight cough, but it had left him, and that now he was entirely well, with the exception of his strength. His general expression was that of malaise, and the only functional symptom present was a difficulty of breathing on taking exercise.—From this time I became a daily visitor of him, and never found him with any increase of symptoms. He was without cough throughout his illness. He could lie with his head high or low, and on either side indiscriminately; had no fever; with a moist skin, and healthy secretions. By means of percussion and auscultation the following diagnosis was formed:—That there was effusion in the left pleura, resulting from a chronic pleuritis, which had extended itself to the substance of the lung. Percussion was dull over the entire left side of the chest. Auscultation detected no respiratory murmur, except near the summit of the lung, and there it was bronchial. In the measurement of the thorax below the nipple, the left side was rather more than an inch the largest. The resonance of the voice was considerable in the upper anterior and posterior portion of the chest, giving rise, (so far as I could discriminate,) to broncophony. I could not detect egophony. The right lung afforded no deviations from a normal condition, with the exception of an increase of its healthy sounds, which I supposed to be owing to a greater demand on that lung to carry on the suspended action of the left one. These were the functional and physical signs as observed in the case. My friend Dr. Mosley was called in consultation, who confirmed the diagnosis. I will not detail the treatment, but will only add, it is that which is usually adopted. His pulse remained natural almost to his last moments. His death took place on the 23d inst. His body was opened on the 24th, in the presence of Dr. Oley. The left lung was smaller and much thinner than natural, and more than two-thirds was completely solidified, the remaining upper portion was partially so. There were no tubercles discovered. The left pleura contained by estimation about three quarts of yellow fluid, resembling serum. Firm and extensive pleuritic adhesions existed; the lower part of the lung adhered to the diaphragm, through adventitious membrane. The right lung seemed entirely normal. The abdominal viscera were healthy. The remainder of the body was not examined.

I should have mentioned the condition of the heart, which was perfectly healthy. Here was a case of insidious, extensive, and fatal disease of the lung, without the patient ever complaining of anything. There was but one functional symptom to give a clue to this mass of disease, and that was a difficulty in breathing when he took exercise. Although in my infancy in the physical mode of examining the chest, yet it must be seen that without the knowledge *I even* possessed, I must have ever remained in the dark as to the true nature of the disease. I might perhaps from his dyspnœa have suspected some disease about the lungs, but of the nature and seat, or even on which side it existed, I never could have known unless aided by acoustics.

*Bedford County*, (*Va.*,) July 26th, 1841.

